# Dieulafoy’s disease of the bronchial tree: a case report: Doença de Dieulafoy da árvore brônquica: relato de caso

**DOI:** 10.1590/1516-3180.2016.0258191116

**Published:** 2017-05-29

**Authors:** Massoud Baghai Wadji, Athena Farahzadi

**Affiliations:** I MD. Associate Professor of Surgery, Firuzgar Hospital, Iran University of Medical Sciences, Tehran, Iran.; II MD. Resident of General Surgery, Iran University of Medical Sciences, Rasool Akram Hospital, Shahrara, Tehran, Iran.

**Keywords:** Dieulafoy disease, Bronchi, Hemoptysis, Pulmonary artery, Lung lobectomy

## Abstract

**CONTEXT:**

Dieulafoy’s disease of the bronchial tree is a very rare condition. Few cases have been reported in the literature. It can be asymptomatic or manifest with massive hemoptysis. This disease should be considered among heavy smokers when recurrent massive hemoptysis is present amid otherwise normal findings. The treatment can be arterial embolization or surgical intervention.

**CASE REPORT:**

A 16-year-old girl was admitted to the emergency department due to hemoptysis with an unknown lesion in the bronchi. She had suffered massive hemoptysis and respiratory failure one week before admission. Fiberoptic bronchoscopy revealed a lesion in the bronchus of the right lower lobe, which was suspected to be a Dieulafoy lesion. Segmentectomy of the right lower lobe and excision of the lesion was carried out. The outcome for this patient was excellent.

**CONCLUSION:**

Dieulafoy’s disease is a rare vascular anomaly and it is extremely rare in the bronchial tree. In bronchial Dieulafoy’s disease, selective embolization has been suggested as a method for cessation of bleeding. Nevertheless, standard anatomical lung resection is a safe and curative alternative.

## RESUMO

**CONTEXTO:**

A doença de Dieulafoy da árvore brônquica é uma condição muito rara, poucos casos foram descritos na literatura. Pode ser assintomática ou manifestar-se com hemoptise maciça. Esta doença deve ser considerada em fumadores pesados quando eles têm recorrentes hemoptises maciças sem outros achados anormais. O tratamento pode ser tanto embolização arterial como intervenção cirúrgica.

**RELATO DE CASO:**

Uma menina de 16 anos foi admitida no Serviço de Urgências devido a hemoptise com uma lesão nos brônquios de origem desconhecida. Havia sofrido hemoptise maciça e insuficiência respiratória uma semana antes da admissão. A broncoscopia de fibra óptica relevou lesão no brônquio do lobo inferior direito, com suspeita de ser lesão de Dieulafoy. Foi realizada uma segmentectomia do lobo inferior direito com excisão da lesão. O resultado da paciente foi excelente.

**CONCLUSÃO:**

A doença de Dieulafoy é uma anomalia vascular rara, sendo extremamente rara na árvore brônquica. Na doença de Dieulafoy bronquial, embolização seletiva tem sido sugerida como método para cessação do sangramento; no entanto, a habitual resseção anatômica do pulmão é uma alternativa segura e curativa.

## INTRODUCTION

Dieulafoy’s disease of the bronchial tree is a very rare disease. Few cases have been reported in the literature. It can be asymptomatic or can manifest with massive hemoptysis. This disease should be considered among heavy smokers with recurrent massive hemoptysis.^1^

The diagnosis can be confirmed by means of bronchoscopy, which shows aberrant arterial bleeding in the bronchial tree. Imaging, consisting of either normal chest X-ray or chest computed tomography (CT) scan, can be helpful in making the diagnosis, through ruling out other causes of hemoptysis.

The treatment usually comprises arterial embolization. If this method is unavailable or unsuccessful, surgery can be another option for achieving a definitive cure.

Here, we report a case of Dieulafoy’s disease in a girl who presented with massive hemoptysis, which was diagnosed by means of bronchoscopy and treated through segmentectomy.

## CASE REPORT

A 16-year-old nonsmoking girl was referred to our hospital because of an episode of massive hemoptysis. She had been admitted to a local hospital one week earlier because of this symptom and had developed respiratory failure, requiring mechanical ventilation for two days. After extubation and cessation of bleeding, she was referred to our hospital for further evaluation.

On admission to the thoracic surgery department, she was conscious and extubated, without respiratory distress, but mildly anxious. Her vital signs were stable and she was afebrile. Physical examination on the head and neck, chest, abdomen and extremities showed that these were normal. Oxygen saturation in the ambient air was 98%. There was no longer any hemoptysis.

**Figure 1.**
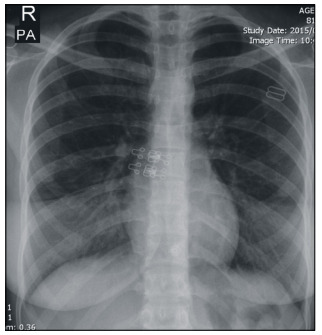
Chest X-ray showing nearly normal lung field.

**Figure 2.**
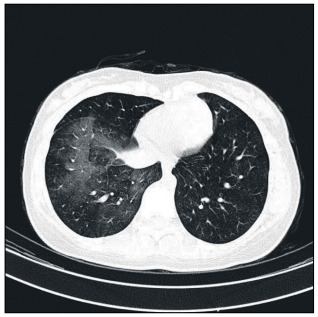
Computed tomography scan of the chest (pulmonary window), depicting patchy alveolar hemorrhage in right lower lobe.

Laboratory data including white blood cell (WBC) and platelet counts, hemoglobin and hematocrit, prothrombin time, partial thromboplastin time (PTT) and international normalized ratio (INR) were within normal limits. A chest X-ray was normal, while chest CT scans showed some patchy haziness in the right lower lobe and a very small lesion in the distal bronchus intermedius (Figures 1 and 2). The imaging did not show any atelectasis, honeycomb appearance, cavitation, consolidation or tumoral lesion. Common causes of massive bleeding like bronchiectasis, carcinoid tumor, tuberculosis, arteriovenous (AV) malformations and other conditions were less likely to be the reason for the bleeding in this girl.

**Figure 3.**
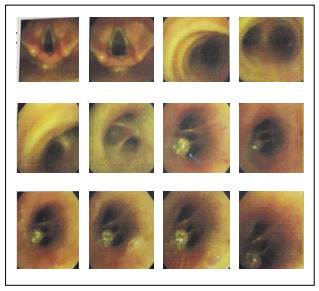
Bronchoscopy showing a small lesion in the bronchus of basal segments of right lower lobe (arrow).

**Figure 4.**
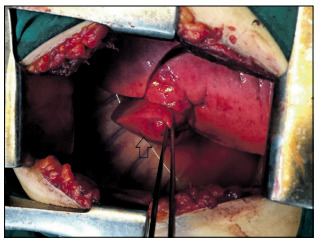
Right thoracic cavity after basal segmentectomy on the right lower lobe. The arrow shows upper segment of right lower lobe.

On the next day, fiberoptic bronchoscopy was performed and this showed a lesion at the beginning of the bronchus of the basal segments of the right lower lobe, without evidence of active bleeding. The lesion originated from the mucosal surface, with a small clot over it. The mucosa surrounding the lesion was absolutely normal (Figure 3). No biopsy was taken, because of the suspicion of Dieulafoy’s disease and the risk of bleeding. Given the lack of expertise in bronchial angiography and embolization at our center, we preferred surgical treatment. Therefore, within an elective setting and after hemorrhaging had ceased, basal segmentectomy of the right lower lobe was carried out in a planned manner, by means of right lateral thoracotomy. The superior segment of the right lower lobe remained intact (Figure 4). There was no intraoperative finding except for consolidation of the parenchyma of the diseased lobe, most probably due to hemorrhage. The operation was performed without any difficulty because of normal anatomical integrity.

An intraoperative frozen section study was negative for any malignant condition.

The patient had a very smooth and uneventful postoperative course, in which she only presented pain, which could be controlled with ordinary analgesics. She was discharged on the sixth postoperative day. At an outpatient visit one week later, she did not have any serious complaint. Moreover, in the third, sixth and eighteenth months of follow-up, she was still asymptomatic without recurrence of any kind of hemoptysis.

**Figure 5.**
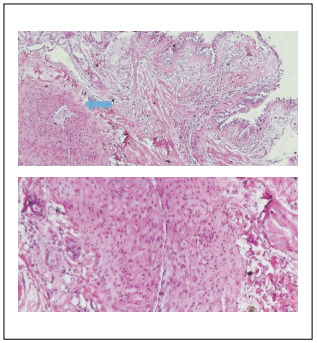
Histological section through bronchial Dieulafoy lesion (arrow: dilated hypertrophic submucosal artery; 2.5 X magnification, hematoxylin and eosin staining).

Although the diagnosis of this disease was clinical, further pathological studies showed few dilated vessels in the submucosa. This was compatible with a diagnosis of Dieulafoy’s disease (Figure 5).

## DISCUSSION

Dieulafoy’s disease is a rare vascular anomaly consisting of a dysplastic artery in the submucosa. It is mostly seen in the gastrointestinal tract and is extremely rare in the bronchial tree. To the best of our knowledge, there are only a few reports of Dieulafoy’s disease of the bronchial tree in the English-language literature (Tables 1 and 2 ^1^^,^^2^^,^^3^^,^^4^^,^^5^^,^^6^^,^^7^^,^^8^). Accordingly, the natural history of this disease and the preferred treatment are not known well. On the other hand, the mortality rate in the absence of any treatment rises to more than 50%.^1^

**Table 1.**
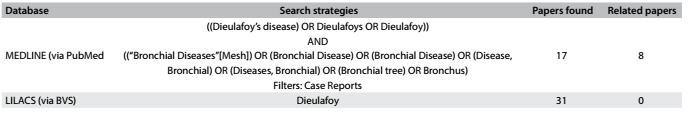
Articles relating to Dieulafoy’s disease that were found through searching the medical literature databases (November 22, 2016)

**Table 2.**
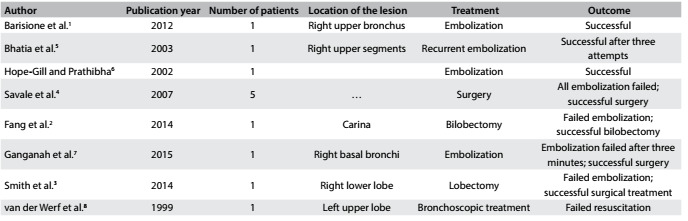
A review of Dieulafoy’s disease reported in the medical literature

The pathogenesis of this disease is also unclear, but most reports state that it occurs in heavy smokers and presents with massive and recurrent hemoptysis. Dieulafoy’s disease of the bronchus may have a congenital origin, arising from either the systemic or the pulmonary circulation.^2^ Spontaneous bleeding has been described in these cases, but bleeding in such cases often occurs after a biopsy on a lesion that has not been diagnosed as a vascular anomaly. Age and tobacco use have an influence on occurrences of this disease.^1^^,^^3^

Dieulafoy’s disease can be suspected when there is severe or massive hemoptysis in the absence of any significant abnormality on either chest X-ray or chest CT scan and in the absence of any medical or surgical history, as in our patient’s case. Bronchoscopy, preferably using a fiberoptic when the bleeding is not severe, may be diagnostic. It will usually make it possible to find both the source and the cause of the bleeding.^3^ The characteristics of the lesion are nonspecific, but it can be suspected when a small (usually less than 1 cm) sessile non-pulsatile nodular lesion with a white cap and apparently normal mucosa is seen.^2^

It has been suggested that, after the diagnosis has been made, angiography and embolization can be the preferred treatment^5^^,^^6^ and that surgical resection would only be needed in a few cases.^4^ However, the failure rate of embolization is not negligible, whereas surgery alone or after failure of embolization has had a success rate of nearly 100% in all reports.^7^ Nevertheless, angioembolization is less invasive than surgery, and both physicians and patients prefer it as the first attempt to halt the bleeding. In the event of surgical intervention, since the lesion is usually located in a lobar or segmental bronchus, the surgery should be carried out as an anatomical segmentectomy or lobectomy. Alternatively, bronchoplastic procedures can be performed if the lesion is located in a major bronchus. There is a lack of long-term follow-up in the reports on patients who have undergone embolization alone.^7^

Although the bleeding recurrence rate in patients whose hemorrhaging has stopped spontaneously is not known, physicians cannot take the risk of not initiating any interventions. If selective embolization is unavailable or if it fails, surgery can be lifesaving. Even in patients whose bleeding stops spontaneously, surgery can have a role in prevention of life-threatening hemoptysis.

## CONCLUSION

Dieulafoy’s disease is a rare vascular anomaly and is extremely rare in the bronchial tree. It should be considered as a diagnosis when there is severe or massive hemoptysis in an otherwise normal patient who has nearly normal chest imaging. Bronchoscopy is diagnostic. In bronchial Dieulafoy’s disease, selective embolization has been suggested as a method for cessation of bleeding. When angioembolization fails or is unavailable, surgical resection consisting of either segmentectomy or lobectomy can be lifesaving for these patients.
